# Invasive Metastatic Well-Differentiated Liposarcoma to the Heart, Found in Intraoperative Transesophageal Echocardiography

**DOI:** 10.7759/cureus.22274

**Published:** 2022-02-16

**Authors:** Junshi Kawakami, Daisuke Sugiyama, Erina Terao, Hiroaki Tanabe, Kenichi Ueda

**Affiliations:** 1 Department of Anesthesiology, Kameda Medical Center, Kamogawa, JPN; 2 Department of Cardiovascular Surgery, Kameda Medical Center, Kamogawa, JPN

**Keywords:** mimicking lipomatous hypertrophy, hematogeneous metastasis, cardio thoracic surgery, transesophageal echocardiography (tee), invasive metastatic well-differentiated liposarcoma

## Abstract

Hematogenous metastasis of liposarcoma to the heart is rare, even though other types of distant metastatic cardiac tumors are relatively more common than primary cardiac tumors. We experienced a case of distant metastasis of liposarcoma to the right interatrial septum, mimicking lipomatous hypertrophy in transesophageal echocardiography (TEE). There were no significant findings in the preoperative transthoracic echocardiography (TTE) or computed tomography (CT). TEE was the only tool to suspect the presence of a cardiac tumor. It also helped evaluate the spread of tumor invasion and make a decision for operation.

## Introduction

Cardiac tumors are rare disease. Although metastatic cardiac tumors are more common than primary ones, hematogenous metastasis of liposarcoma to the heart is extremely rare. Among its variants, well-differentiated liposarcoma is reported to have an infrequent possibility of distant metastasis [1]. Cardiac tumors can be diagnosed due to symptoms or they may be found accidentally during tests for unrelated medical problems. Thus, it is sometimes challenging to identify nonsymptomatic cardiac tumors prior to heart surgery if they are located at blind spots where scanning with transthoracic echocardiography (TTE) is difficult or if they are too small to detect with computed tomography (CT). We experienced a rare case of distant metastasis of liposarcoma to the right interatrial septum, mimicking lipomatous hypertrophy in transesophageal echocardiography (TEE). The patient had provided written consent to publish this case report, and the Research Ethics Committee of Kameda Medical Center approved this case. The case report adheres to the applicable Enhancing the Quality and Transparency of Health Research guideline.

## Case presentation

A 71-year-old woman with hypertension, diabetes mellitus, and hyperlipidemia was scheduled to undergo an elective aortic arch replacement for an aortic arch aneurysm. The aneurysm was found by chance with CT while assessing a sudden loss of consciousness and had expanded by 8 mm diameter while being followed for half a year. The patient had a medical history of adrenalectomy for primary liposarcoma 16 years earlier with no subsequent signs of recurrence. As a part of preoperative workup, TTE was performed, which showed a small high echo density mass in the interatrial septum, considered to be fatty infiltration.

**Figure 1:**
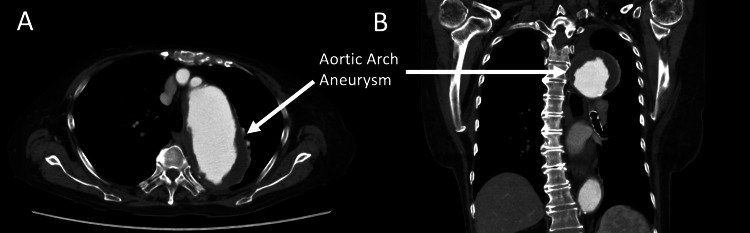
The preoperative computed tomography image. (A) Axial section. (B) Coronal section. The arrows indicate an aortic arch aneurysm with a diameter of 61 mm.

The TTE also showed normal left ventricular function with an ejection fraction of 58% and mild aortic, tricuspid, and pulmonary regurgitation. The preoperative CT also revealed an aortic arch aneurysm with a diameter of 61 mm (Figures 1A, 1B).

**Figure 2:**
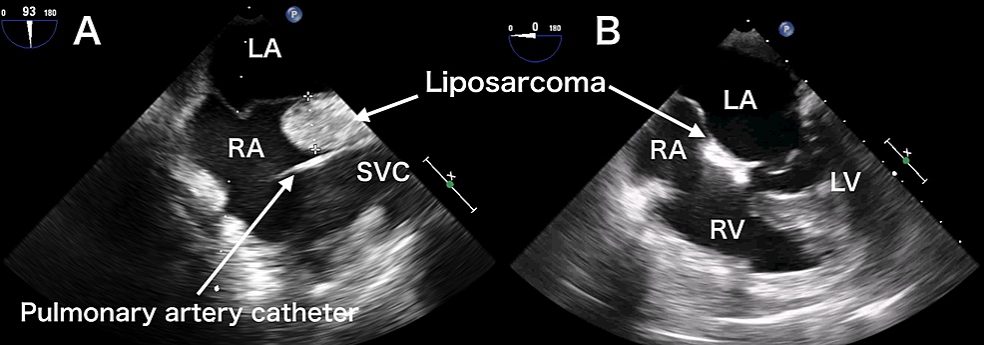
Transesophageal echocardiography during surgery. (A) The bicaval view image showed a finding similar to hypertrophic lipomatous atrial septum, found as a fatty infiltrated structure. (B) The four-chamber view showed that the tumor invaded into the right ventricle. LA: left atrium, LV: left ventricle, RA: right atrium, RV: right ventricle, SVC: superior vena cava.

The radiology did not report any other abnormalities, including heart structure. The electrocardiogram study was read as normal as well. In addition to American Society of Anesthesiologists (ASA) standard monitors, cerebral oximetry, a radial arterial catheter, and pulmonary artery catheter were placed for intraoperative monitoring. General anesthesia was induced with midazolam (3 mg) and fentanyl (300 µg), and tracheal intubation was facilitated by rocuronium (50 mg). Continuous infusion of propofol, remifentanil, and rocuronium was administered for maintenance. After the induction of general anesthesia, TEE examination was performed as a routine prebypass workup. In the midesophageal (ME) bicaval view, abnormal thickening of the interatrial septum extending toward the right atrium (RA) was noted, which was similar to a “dumbbell shape”, lipomatous hypertrophy (Figure 2A).

However, in an ME four-chamber view, the abnormal thickening seemed to extend into the interventricular septum, and the right ventricle (RV) appeared as a high-density echocardiographic structure (Figure 2B). These findings were communicated to the surgical team as they were preparing cannulation for cardiopulmonary bypass. After careful inspection of cardiac structure, they found thickened tissue extending to superior vena cava (SVC), RA, right atrial appendage, and left atrium (LA). Due to the patient's medical history of liposarcoma requiring the previous adrenalectomy, distant metastasis to the heart was suspected and a biopsy of the RA appendage was sent to the pathology laboratory. Before the biopsy, the surgery was suspended, and the patient's family was informed of the situation and consented to a new biopsy. The provisional pathological diagnosis was of a malignant tumor, suspected of being liposarcoma. Considering TEE finding of the tumor invasion into RV, the surgical team judged that the tumor resection would not be possible. The operation was canceled, and the patient was transferred to the surgical intensive care unit (ICU). The immunohistochemistry determined that the tissue was a well-differentiated liposarcoma (WDL), an atypical lipomatous tumor. The oncologist planned for the provision of palliative care to the patient, who was discharged from the hospital on postoperative day 10 without sequela.

## Discussion

The current case highlights the critical role of intraoperative TEE during cardiac surgery in a case where a cardiac tumor was not suspected preoperatively. All past follow-up tests for recurrence had been negative. In addition, there were no significant findings noted from the preoperative TTE and CT. Thus, the recurrence of the tumor to the heart was not suspected preoperatively. Only intraoperative TEE findings allowed us to suspect the tumor metastasis to the heart, which changed the entire surgical plan. Without TEE findings, the surgical team might have proceeded with aortic arch replacement. Although we could not diagnose a cardiac tumor from the findings alone, TEE was helpful to identify the presence of a possible cardiac tumor and to assess invasion to other parts of the heart.

Hematogenous metastasis of liposarcoma is rare among metastatic cardiac tumors. Although the frequency of primary cardiac tumors is 0.1%, metastasis to the heart and pericardium was found to be 8.4% in a series of cancer patient autopsies. However, only a few cases of metastatic liposarcoma have been reported [2]. According to the World Health Organization classification, soft tissue neoplasm has more than 100 histologic subtypes, and liposarcoma is one of the most common subtypes of sarcoma [3]. Liposarcoma develops from adipocyte precursors and is typically found in the extremities and retroperitoneum. It has some variants, with a wide range of histologic differences and biological behavior, including anatomic distribution and a risk of metastasis. Among the variants, WDL, an atypical lipomatous, is the most common subtype, which shows a five-year survival probability of 90%. WDL has a low recurrence rate, and distant recurrence is quite rare. Singer et al. reported that the probability being free of local recurrence at three years for WDL was 69% (58%-81%). The probability of free distant recurrence within three years for WDL was 99% (96%-100%) [1].

Depending on the tumor’s size and location, cardiac tumors can cause various symptoms, including heart failure, myocardial infarction, pulmonary embolism, tamponade with pericardial effusion, arrhythmia, and syncope. However, these symptoms are not specific, and some patients have no symptoms or only minor ones, such as cough, chest discomfort, and peripheral edema [4-6]. Therefore, the diagnosis of cardiac tumors based on symptoms alone can be challenging. Regarding diagnostic examination, the tumor can be identified with TTE, TEE, CT, or magnetic resonance imaging (MRI). When cardiac tumors are suspected, MRI is a gold standard examination, especially for diagnosing tumor etiology by evaluating signal intensity changes on the various sequences [7]. Between TEE and TTE, TEE is superior ultrasound examination for detecting intracardial tumors [4].

## Conclusions

In conclusion, we report the rare case of a distant recurrent liposarcoma to the heart without specific symptoms or clinical findings. Even in such a case, TEE is useful for detecting a cardiac tumor. It also helps evaluate the invasion of tumors and make an operative decision during surgery.

## References

[1] Singer S, Antonescu CR, Riedel E, Brennan MF (2003). Histologic subtype and margin of resection predict pattern of recurrence and survival for retroperitoneal liposarcoma. Ann Surg.

[2] Silvestri F, Bussani R, Pavletic N, Mannone T (1997). Metastases of the heart and pericardium. G Ital Cardiol.

[3] Karanian M, Coindre JM (2015). Fourth edition of WHO classification tumours of soft tissue. (Article in French). Ann Pathol.

[4] Engberding R, Daniel WG, Erbel R (1993). Diagnosis of heart tumours by transoesophageal echocardiography: a multicentre study in 154 patients. Eur Heart J.

[5] Porres-Aguilar M, De Cicco I, Anaya-Ayala JE, Porres-Muñoz M, Santos-Martínez LE, Flores-García CA, Osorio H (2019). Cardiac metastasis from liposarcoma to the right ventricle complicated by massive pulmonary tumor embolism. Arch Cardiol Mex.

[6] Wang Y, Liu S, Yang J, Gu T, Zhang L (2017). Cardiac hemangioma caused ventricular arrhythmia: a rare case and literature review. J Electrocardiol.

[7] Gulati G, Sharma S, Kothari SS, Juneja R, Saxena A, Talwar KK (2004). Comparison of echo and MRI in the imaging evaluation of intracardiac masses. Cardiovasc Intervent Radiol.

